# Differences in Multi-Dimensional Well-Being Among Factory Workers: Evidence from Six Countries

**DOI:** 10.1007/s11482-023-10181-0

**Published:** 2023-05-25

**Authors:** Piotr Bialowolski, Matthew T. Lee, Dorota Weziak-Bialowolska, Ying Chen, Richard G. Cowden, Eileen McNeely, Tyler J. VanderWeele

**Affiliations:** 1 Department of Economics, Kozminski University, Warsaw, Poland; 2 Human Flourishing Program, Institute for Quantitative Social Science, Harvard University, Cambridge, MA, US; 3 Institute for Studies of Religion, Baylor University, Waco, US; 4 Center for Evaluation and Analysis of Public Policies, Faculty of Philosophy, Jagiellonian University, Kraków, Poland; 5 SHINE, Human Flourishing Program, Institute for Quantitative Social Science, Harvard University, Cambridge, MA, US

**Keywords:** Social relationships, Financial security, Health, Meaning and purpose in life, Character and virtue, Well-being

## Abstract

This paper presents cross-cultural comparisons of well-being among factory workers, as measured by the six well-being domains of happiness and life satisfaction, physical and mental health, meaning and purpose, character and virtue, close social relationships, and financial and material stability. Relative ranks of well-being domains across examined groups of workers are also compared. Results are based on survey data from factory workers in Cambodia, China, Mexico, Poland, Sri Lanka, and the United States. Average well-being scores are higher among factory workers in Mexico, China, and Cambodia than in the U.S., Poland, and Sri Lanka across all domains except financial and material stability. Close social relationships were the highest ranked domain in Cambodia and China but ranked much lower (5th) in the U.S. Meaning and purpose, as well as character and virtue were highly ranked across the board. Strong social relationships seem to thrive in contexts where financial insecurity is high.

## Introduction

Demand for labor generated by global brands in emerging economies led to increased job opportunities for local workers (Barrientos, Gereffi, et al. 2011; Barrientos, Mayer et al. 2011). Ensuring appropriate working conditions and welfare of factory workers, however, has been rarely an important issue in the process of work-post creation (Egels-Zandén & Lindholm, 2015). Especially in less developed countries, where insufficient rule of law accompanied by flawed legal systems are present, working conditions can lead to various negative outcomes in the life domain (Węziak-Białowolska et al., 2020). There has already been research demonstrating that in factories with insufficient access to basic sanitation and health care services (CSDH 2008; Marmot et al., 2008), as well as generating strain on work-life balance (Bambra et al., 2014; de Neve et al., 2018), detrimental effects on well-being of workers can be observed. Despite existing evidence that promoting worker well-being can be financially beneficial (Bialowolski et al., 2020), companies are often reluctant to improve standard-of-care for their workers (Adler et al., 2017; Arnold, 2014; (Brown et al., 2014a, b).

The well-being of low-skilled workers is often a low priority target for employers. Such workers are comparatively easy to replace and, consequently, there are lower costs associated with their recruitment, compensation, on-boarding, and training compared to high-skilled workers. However, as labor markets in the developing world mature, buyers and suppliers have become more concerned about difficulties in acquiring workers. In the Chinese economy, for example, constant growth over the past three decades intensified the competition for labor at all levels of skills (Zhang et al., 2011). Low well-being can potentially exacerbate the problem of scarcity of workers as it often translates into high turnover rates (De Croon et al., 2004; Holtom et al., 2008) and low productivity (Levi et al., 2022).

In this study we examine the well-being of production workers. We offer a comparison of well-being that includes both psychosocial and material dimensions (Lee et al., 2021), which can help to identify dimensions of well-being in which production workers have the largest distance to catch-up, as well as highlight the well-being strengths. This study is also in line with a number of worker well-being programs that promote worker well-being on and off the job. They include the Total Worker Health^®^ program by United States Centers for Disease Control and Prevention’s National Institute for Occupational Safety and Health (CDC/NIOSH) (Schill, 2017; Tamers et al., 2019), the Model for Action by WHO (2010), and the Better Work program by the United Nation’s International Labour Organization (ILO) and the World Bank’s International Finance Corporation (IFC)^1^.

Our analysis is drawn from large samples of employees of five apparel companies that launched a worker well-being program, located in Cambodia, China, Mexico, Poland, Sri Lanka, and two manufacturing companies in the United States that also implemented a worker well-being initiative. Our results do not generalize to all members of the respective societies but particularly focus on production workers. Focusing on the well-being of working adults employed in production companies is especially important because this group generates considerable value added that sustains other social groups (i.e., families and communities), while at the same time they are a group at high risk of turnover because of the precarity of their employment. Additionally, evidence from prior studies suggests that greater worker well-being is positively associated with work-related functioning. For example, longitudinal studies have shown that happiness and life satisfaction are associated with higher subsequent job happiness and job satisfaction, meaning in life is associated with greater subsequent meaning at work, and psychological climate for caring at work is associated with higher subsequent work productivity and work quality (Weziak-Bialowolska et al., 2020, 2023). Other longitudinal research demonstrated that better social well-being is positively associated with work productivity (Bialowolski et al., 2020), while physical health and mental health problems contributed to increased dysfunctional presenteeism (Bryan et al., 2022).

## Challenges in Measurement of Well-Being

Historically, anthropologists, seeking to avoid ethnocentrism, have been reluctant to evaluate the overall well-being of a group using standards that originate outside of that group. Comparisons are also complicated by cultural variations in language, the role of memory in determining subjective well-being, and the presence of a positivity or social desirability bias (Biswas-Diener & Diener, 2001; Diener et al., 2017; Fisher & Katz, 2000; Oishi, 2002). Pancultural aspects of well-being have been also identified in self-report studies. For example, survey researchers provided a support for concepts such as a “livability” to describe “conditions of quality of life in societies that meet human needs” and which arguably reflect “universal human conditions that will lead to well-being” (Diener, 2009, p. 2). Prosocial behavior has also been shown to positively impact self-reported positive affect around the world (Jebb et al., 2020).

Beyond findings on the individual domains of well-being, recent conceptual and empirical scholarship has established a flourishing concept composed of at least five core well-being domains which are “nearly universally desired” and widely considered as ends in themselves (VanderWeele, 2017). They include happiness and life satisfaction, mental and physical health, meaning and purpose, character and virtue, and close social relationships. A sixth domain— financial and material stability—may be necessary to sustain well-being in the other domains over time. Including this additional domain allows us to assess *secure* flourishing (VanderWeele, 2017; Weziak-Bialowolska et al., 2019a). There is also some evidence that these domains are “culturally universal” (Weziak-Bialowolska et al., 2019b, p. 9).

Various self-report measures of well-being, have been developed, including the Mental Health Continuum–Short Form (Keyes, 2002), the well-being items on the European Social Survey (Huppert et al., 2009), the Flourishing Scale (Diener et al., 2010), and the PERMA-Profiler (Seligman, 2011). Although they encompass multiple dimensions of well-being and have made important contributions to our knowledge base, these measures tend to omit physical health, as well as many aspects of character strengths and financial security.

Cross-cultural comparisons to date—using data from such surveys as the Gallup World Poll, the World Happiness Survey, or the World Values Survey—often focus on a somewhat restricted subset of well-being measures, either physical health, financial security, subjective well-being (SWB), or selected aspects of SWB such as happiness or life satisfaction (cf. Helliwell et al., 2019; Jebb et al., 2020). The European Social Survey provides more comprehensive information on cross-cultural well-being but is limited to European countries and missing on highly valued domains, as we have already noted (Hone et al., 2014). A general conclusion of the existing research is that most people throughout the world report “moderately positive” SWB (Diener & Suh, 1999, p. 435). Furthermore, in some studies individualism predicts SWB and is also correlated with income, such that positive economic development seems to foster well-being in general (Diener, 2009; Diener et al., 2017; Diener & Suh, 1999) and its financial dimension (Xiao & Bialowolski, 2023), although higher income does not necessarily increase well-being longitudinally (Easterlin et al., 2010). Attempts to make general statements are also complicated by findings suggesting that countries that are materialistically successful, boasting the highest rates of “happiness” and personal security, also have high levels of “deaths of despair” (i.e., suicide, drug overdose, cirrhosis of the liver; Case & Deaton 2015) and a sense of meaninglessness (Froese, 2016; Oishi & Diener, 2014), at least among some strata of their populations. Such countries may lack the “sacred canopies and existential urgency” that has historically seemed to “guarantee a meaningful life” (Froese, 2016, p. 54; Lee et al., 2019; Smith, 2017). Paradoxically, “reported SWB and suicide correlate across nations” (Diener & Suh, 1999, p. 442). In the struggle to survive in economically under-developed contexts, connections to both horizontal (other people) and vertical (higher power) “significant others” are strong. Therefore, respondents from wealthy countries may score higher on some aspects of well-being like SWB, but simultaneously report lower levels of spiritual well-being, social connectedness, and sense of purpose (Froese, 2016). Significant social problems may be underappreciated with pursuing a more narrow idea of well-being, highlighting the value of a more holistic appraisal of complete wellbeing (see Lee, Kubzansky, et al. 2021).

## Cultural Differences in Domains of Well-Being

Previous surveys have revealed that eight out of ten respondents throughout the world are “very or quite happy”; that wealth, human rights, political stability, and other markers of material security are generally good predictors of increased well-being across nations; and that the happiness of individuals within and across countries varies over time (Diener et al., 2006, p. 3; Helliwell et al., 2019; Jebb et al., 2020). Despite the reportedly high levels of *happiness*, data from the Gallup World Poll suggest that less than a quarter of the world’s population is “thriving” according to a combination of current and future *life evaluation* ratings, that the global average score for current life evaluation is only a 5.3 out of a maximum of 10, and that there has been a dramatic rise in negative emotions around the world in recent years, especially in countries that have experienced social and political upheavals (Clifton, 2022). Such variations across time and place challenge the “automatic habituation model” (aka the “hedonic treadmill”) that people adapt to their environments by returning to a “set point” of neutral well-being regardless of contextual differences (Diener et al., 2006, p. 2; Headey 2008). Instead, a great deal of cross-national variation has been observed, not only for happiness but for other measures of flourishing as well (Helliwell et al., 2019; Myers & Diener, 1995). For example, an examination of dichotomized hedonic and eudaimonic aspects of well-being across European nations revealed a four-fold difference between the highest and lowest scoring countries (Huppert & So, 2013). With regard to the factors that shape well-being across cultures, some studies support the *universalist approach* that there are culturally invariant needs driving well-being, such as competence, autonomy, and relatedness (Deci & Ryan, 2000); others support the *cultural approach* which argues for variation (Krys et al., 2023); and finally, there is the *mixed approach* which suggests the existence of culturally invariant needs but posits that the importance of these needs may vary across societies (Ng & Diener, 2014).

The very meaning of “happiness” diverges across cultures, with “feeling good about oneself” (Diener, 2009, p. 4; Diener & Suh 1999) being more highly prized in individualistic nations than collectivist ones and self-esteem also serving as a stronger predictor of life satisfaction where individualism is high (Diener et al., 2017). Moreover, relationship satisfaction is more important for SWB in collectivist countries, as well as those that are less economically developed (Galinha et al., 2016). Some studies have found that financial satisfaction is more strongly associated with life satisfaction in poorer nations (Diener, 2009), while other research reveals the opposite (Ng & Diener, 2014). This distinction between the *material dimension* of well-being (e.g., physical health and financial security) and the *psychosocial dimension* (e.g., emotional health, social connectedness) has proven helpful for understanding demographic differences in valuing the various domains of well-being (Diener & Suh, 1999; Lee et al., 2021a). Cross-cultural research concludes that although “material luxuries” (Diener, 2009, p. 7) are not necessarily required for a minimal level of well-being in every culture, it is clear that countries that better meet both the material and non-material needs of their populations, generally have higher SWB than those that are less able to meet both of these types of needs (Ng & Diener, 2014; Welzel & Inglehart, 2010).

Beyond this cultural universal, there are significant foundational differences in well-being in cultures derived from the Aristotelian tradition in the West. They tend to stress “analytic (oppositional) thinking,” autonomy, self-assertion, independence, and individual rights. Those with a Confucian heritage in the East are more likely to promote “holistic (dialectical) thinking,” interdependence, harmony, and equanimity (Nisbett et al., 2001; Steger et al., 2008, p. 663). For example, balance, contentment, and other low-arousal emotions are more highly regarded in Eastern cultures, whereas excitement and other high-arousal emotions are esteemed in Western cultures like the U.S. (Diener et al., 2017; Kitayama et al., 2010). This may account for higher levels of SWB in the West (Myers & Diener, 1995), although an assessment of well-being might lead to different results. Similarly, North Americans have a largely positive view of ‘happiness’—more is better—whereas some groups of Asians may worry about “too much happiness” (Diener et al., 2017). In addition to the East/West distinction, there appears to be a pan-Latino effect on SWB, as Latin American countries with a shared history and culture exhibit higher scores than other countries with a similar level of economic development (Ortiz-Ospina & Roser, 2017). There is also abundant evidence of racial and ethnic minorities within countries exhibiting better-than-expected well-being, such as emotional and physical health, despite exceedingly adverse social conditions, which may be explained by cultural norms that foster higher levels of social connections (Gõmez-Puerta et al., 2015; Keyes, 2009; Lee & Martinez, 2006; Ryff et al., 2004).

In other words, the structural factors that are often posited to account for variations in well-being across groups, such as material wealth, are frequently moderated by cultural factors. One study of people living in deplorable conditions in Calcutta, India (in slum housing, brothels, or on the streets), found that their life satisfaction is higher “than one might expect” and this outcome was related to a “strong emphasis on social relationships and the satisfaction derived from them” (Biswas-Diener & Diener, 2001, p. 261). Cultures that value social relationships highly, and this is particularly true in collectivist and economically underdeveloped countries like India (Galinha et al., 2016), enable impoverished people to “utilize their strong social relationships” to partially offset the threats to well-being that are posed by poverty (Biswas-Diener & Diener, 2001, p. 275). More generally, if an individual’s psychosocial orientation fits well with the values promoted by the “dominant” culture in that individual’s society, then they are more likely to experience a higher degree of flourishing. For example, religious people experience higher levels of happiness generally, but this is most the case in religious nations rather than in nonreligious nations (Diener et al., 2017; Oishi & Diener, 2014; Pawlikowski et al., 2019). To relate this to the culture of Calcutta, outside observers who expect low well-being due to extreme poverty may overlook important positive cultural aspects: a high sense of moral goodness, religiosity, social connectedness, and family ties (Biswas-Diener & Diener, 2001). An individual who values these aspects of the culture will flourish in Calcutta to a much greater degree than one who exalts materialistic ideals.

## Challenges in Cross-Cultural Comparison

Our examination of production workers from one of the most developed countries (two U.S. samples) and two other developed countries (Poland and Mexico, according to OECD) provide the benefit of benchmarking relative to other examined samples including those from Sri Lanka, Cambodia, and China. However, since our samples are from culturally different societies, comparing them in terms of well-being presents a number of challenges, including (but certainly not limited to) socially desirable responding, acquiescence, extreme response bias, and difficulty translating words across cultures (Fisher & Katz, 2000; Morren et al., 2012; van Herk et al., 2004). We review a few examples of such complexities, which underscore the caution required in comparing absolute levels of the domains of well-being across countries. We therefore generally focus our discussion on the relative ordering of domains. We have already noted that cultures place different levels of value on specific aspects of well-being such as happiness. But in addition to the East/West cultural divide, there are also different thresholds for being ‘happy’ within Western cultures, with the word being used much more liberally in the English language than in French and German (Diener et al., 2017). It is not particularly informative to simply ask about levels of happiness and compare results across societies without understanding cultural variations in using this word and its translations. Other words are similarly polysemous within and across cultures (Ravin & Leacock, 2000).

Lexical variation is not the only problem. There are also biases with regard to how emotions, and well-being more generally, are remembered (Oishi, 2002). In some cultures, the accuracy of a respondent’s assessment of their emotional state over the past week depends on their “general emotional self-perception” (Diener, 2009, p. 6), whereas in other cultures this kind of bias is less evident. This is especially important for conceptions of well-being that include the ratio of positive to negative emotions. Negative affect is more normal in some cultural traditions than others, while specific aspects of well-being such as close relationships or financial security may generate negative feelings as well as happy ones (Myers & Diener, 1995). Emotions like pride cluster with positive emotions depending on the culture, while love might be related to sadness in some cultures but not others (Diener, 2009). Cross-cultural differences with regard to a “positivity bias” may also shape survey results, as revealed by a comparison of the “evenhanded” manner in which Indians evaluate all domains of SWB, while Americans seem to concentrate “primarily on their best areas” (Biswas-Diener & Diener, 2001, p. 274).

Nevertheless, an examination of the ordering of the domains within and across contexts may be of considerable interest, even if mean scores themselves are not always directly comparable. However, findings presented by Weziak-Bialowolska et al. (2019b) offer some reassurance that with the measurement invariance established for well-being domains (as well as satisfactory reliability and criterion validity), the universal character of the indices and the comparability of their latent scores in five culturally distinct populations examined in this study (except Poland) is largely substantiated. Evidence supporting valid and reliable interpretation of scores on the multidimensional measure of well-being has also been found in numerous workplace settings (Weziak-Bialowolska et al., 2019b) and in multiple cultures in different parts of the world (Höltge et al., 2022). Furthermore, observing means in each domain across different settings can be useful for benchmarking purposes in interpreting future data collection efforts and summaries.

## Materials and Methods

### Participants

Participants were production workers from Cambodia, China, Mexico, Poland, Sri Lanka, and the United States. Data collection took place between 2017 and 2019 (exact dates are presented in Table 1). The largest sample was available from Mexico and comprised 2,723 factory workers. In China, Sri Lanka and the U.S. more than one thousand responses were collected (1,272, 1,284, and 1,268 respectively). The remaining samples were smaller but still substantial – Poland (615) and Cambodia (572).

**Table 1:** Basic Statistics

	Sri Lanka	Mexico	China	Cambodia	Poland	U.S.
Females (%)	57.7	47.6	75.8	86.7	70.2	16.4
Age - mean (SD)	30.6 (9.2)	34.9 (10.6)	35.4 (9.2)	24.6 (4.9)	43.3 (7.8)	45.4 (12.1)
Having children under 18 years old who currently live with the interviewed worker (%)	46.2	76.2	85.0	56.3	61.7	42.6
Married (%)	58.1	40.2	84.0	60.1	74.5	69.0
Education (at least high school) (%)	90.3	82.7	24.0	19.2	92.7	99.0
Number of workers surveyed	1284	2723	1272	572	615	1268
Response rate	96.8%	96.4%	98.0%	94.5%	98.3%	99.3%
Date	Aug 2017	Apr 2019	Dec 2017 – Jun 2018	Nov 2019	Feb 2019	Jun 2018 – Nov 2018

Note: unweighted data

Participants were factory employees of either apparel (non-U.S. samples) or manufacturing (U.S. samples) companies that had a worker well-being program implemented and were interested in examining and improving well-being of their employees. Research team was approached by these companies with an official request to pursue an examination of worker well-being.

### Procedure

All participants received the Worker Well-Being Survey (WWBS) tool (reference blinded for review). This survey questions designed to measure various aspects of worker well-being in addition to physical working conditions, psychosocial job demands and resources, healthcare resources at work, and supportive social and physical resources in the community and within the respondent’s household. It also includes a set of self-reported work performance outcomes, such as self-reported work injury, work quality, absenteeism, as well as job attitudes such as job satisfaction and work engagement. In the current study the focus is solely on well-being. 

The WWBS is used in culturally and linguistically diverse environments. The research team strived to ensure cross-cultural comparability of question wordings, as well as their appropriateness in light of diverse literacy levels. Questions were originally in English but for the non-U.S. samples they were translated from English to specific languages by professional translators. Back-translation was completed by English-speaking students at Harvard University, as well as independent translators from the countries where research was conducted. Discrepancies between translations and the original items were discussed within the research team. A Community Advisory Board was created for each country, comprised of English-speaking citizens of the country where the research was planned and who had appropriate professional expertise in well-being research. They revised the English and country-specific versions of questions based on their appropriateness (understanding and perception) for the relevant worker population. Finally, before survey administration, 10–12 selected workers at each organization working at the most entry-level positions took the survey and discussed the issues related to question comprehension with the research team. The final revisions to the survey were introduced on site to ensure understanding of the survey questions, their appropriateness for the specificity of surveyed populations and to avoid cultural bias, while at the same time maintaining their comparability across cultures.

Survey data were gathered directly at the factories. A communication campaign took place prior to the survey in order to invite and encourage workers to participate. Responses were collected on the Qualtrics platform using a tablet app. Participation rates were between 96% and 99% (Table 1). During the survey administration, groups of workers were released from their positions (most often one production line at a time) to come to the survey stations. Workers’ decision to participate in the survey was voluntary and was not disclosed to management. Remuneration was not affected. In particular, for the time spent on the survey completion workers were paid a piece rate corresponding to their average wage. Confidentiality of survey responses was also ensured as individual survey responses were never shared with factory management. Aggregate survey results were provided to both participants and management on separate occasions.

### Measures

The Secure Flourishing Index was used to examine six dimensions of well-being (VanderWeele, 2017; VanderWeele et al., 2019). This instrument exhibits good psychometric properties while applied in workplace settings (Weziak-Bialowolska et al., 2019a, b; alpha = 0.89 without financial items or alpha = 0.86 with financial items). It assesses the following six domains of well-being with a total of twelve items, scored zero to ten: *happiness and life satisfaction, mental and physical health, meaning and purpose, character and virtue, close social relationships*, and *financial and material stability*. We averaged these six domains to create a comprehensive well-being measure *Secure Flourish Index* (SFI) and we averaged the first five (excluding financial and material stability, which may not be an end in itself) to create a restricted well-being measure *Flourish Index* (FI).^2^ Although the word “flourish” was used in the name of the measure, and we are retaining the name of the measure to be consistent with previous research, for reasons mentioned above we see this as a measure of well-being (see VanderWeele & Lomas 2022).

### Statistical Analysis

Basic sociodemographic characteristics of the examined samples were presented (Table 1). A comparison of the samples revealed dissimilarities. To account for these discrepancies, post-stratification weights were calculated. To this end, a weighting algorithm based on an iterative proportional fitting (raking) was applied (Deville et al., 1993; Kolenikov, 2014) to calculate the weights (raked weights). The following characteristics were included in the process: gender, age group, and marital status, and the Mexican sample was established as a reference sample as it was the largest. The algorithm implemented in Stata 17 converged. The weights ensured that the marginal proportions of respondents with respect to gender, age, and marital status in each sample matched the respective proportions observed in the Mexican sample. In other words, with the raked weights applied, all samples were comparable in terms of basic demographics.

Mean levels of SFI, FI, and all domains were calculated using weighted data. Average levels were compared using one-way ANOVA analysis and the pairwise comparisons were conducted (with the weights applied). Sidak correction was applied to account for multiple testing.

## Results

Table 2 presents our main results, including the average scores (and standard deviation) for each well-being domain, the FI and the SFI, along with the within-sample rank ordering of each domain, across selected production organizations in six countries. Although we report absolute scores with caution because of semantic variations across cultures, potential cultural differences in response styles to items on the FI and SFI, and sociodemographic differences in the samples (partially accounted for with applied weighting scheme), it is interesting to note that average FI scores are more than one point higher in Mexico, China, and Cambodia than in the U.S., Sri Lanka, and Poland and domain scores in these three countries are higher across all domains except financial and material stability. For example, Cambodia’s average FI (8.86) was the highest of any survey, while the U.S. average FI score was 7.34. The position of the U.S. relative to the three countries with the highest average FI shows marginal improvement when the domain of financial and material stability is included, as the highest SFI score (Cambodia at 8.17) is slightly more than 0.9 point higher than the U.S. SFI score (7.26). In some countries, production workers included in this study appear to have very low financial and material stability, with Mexico (3.06) and Poland (3.83) scoring the lowest. However, a low score on this domain is not necessarily related to low scores on other domains. In other words, financial stability is not required for a high level of overall well-being.

**Table 2:** Flourishing, Secure Flourishing, and Domains of Flourishing by Country

Well-being	Sri Lanka (1)		Mexico (2)		China (3)		Cambodia (4)		Poland (5)		U.S. (6)		p-value for F-test	Pairwise comparisons (pairs with not significant difference; p < 0.05)
	Mean (SD)	Rank	Mean (SD)	Rank	Mean (SD)	Rank	Mean (SD)	Rank	Mean (SD)	Rank	Mean (SD)	Rank		
Happiness and Life Satisfaction	6.39 (3.13)	5	8.29 (1.97)	5	8.07 (2.10)	5	8.69 (1.29)	4	7.01 (1.65)	5	7.28 (1.79)	4	< 0.001	(5) vs. (6)
Mental and Physical Health	7.54 (2.50)	3	8.68 (1.54)	2	8.73 (1.66)	2	8.48 (1.44)	5	7.10 (1.78)	4	7.29 (1.67)	3	< 0.001	(2) vs. (3), (2) vs. (4), (3) vs. (4), (5) vs. (6)
Meaning and Purpose	7.99 (2.49)	2	9.16 (1.46)	1	8.27 (1.87)	4	8.91 (1.21)	3	7.52 (1.76)	2	7.37 (2.03)	2	< 0.001	(5) vs. (6)
Character and Virtue	8.03 (2.71)	1	8.64 (1.56)	3	8.38 (1.90)	3	9.05 (1.33)	2	8.15 (1.41)	1	7.73 (1.59)	1	< 0.001	(3) vs. (5)
Close Social Relationships	7.52 (2.86)	4	8.62 (1.94)	4	8.85 (1.62)	1	9.18 (1.06)	1	7.36 (2.03)	3	7.06 (2.17)	5	< 0.001	(1) vs. (5), (5) vs. (6)
Financial and Material Stability	5.84 (3.20)	6	3.06 (3.19)	6	6.13 (3.35)	6	4.78 (3.46)	6	3.83 (2.82)	6	6.85 (2.72)	6	< 0.001	(1) vs. (3)
Flourish Index	7.43 (2.13)	na	8.67 (1.23)	na	8.47 (1.40)	na	8.86 (0.95)	na	7.42 (1.34)	na	7.34 (1.50)	na	< 0.001	(1) vs. (4), (1) vs. (5), (1) vs. (6), (5) vs. (6)
Secure Flourish Index	7.18 (1.89)	na	7.75 (1.26)	na	8.07 (1.43)	na	8.17 (1.02)	na	6.82 (1.22)	na	7.26 (1.49)	na	< 0.001	(1) vs. (6), (3) vs. (4)
N	1284		2723		1272		572		615		1268			

Note: SD – standard deviation; p-value for F-test in one-way ANOVA; weighted results (gender, age, marital status); Sidak correction for multiple testing was applied for pairwise comparisons

Between-sample comparisons (Table 2, last two columns) showed that there are differences between all examined samples in terms of all well-being domains, the FI, and the SFI (in each case, p-value for the F-test in one-way ANOVA was < 0.001). Pairwise comparisons indicated that the mean scores were most heterogeneous for the domain of happiness and life satisfaction (the only similar pair was the U.S. and Poland), meaning and purpose (the only similar pair was the U.S. and Poland), character and virtue (the only similar pair was China and Poland), and financial and material stability (the only similar pair was Sri Lanka and China). Mean scores were most homogeneous on the domain of mental and physical health and the FI (four pairs of similar samples were observed in each case).

We now turn to the rank ordering of the domains observed in Table 2. Social connectedness was the highest ranked domain in two countries with the highest levels of overall well-being (see the mean SFI scores in Cambodia and China), but it was ranked much lower in the U.S., Mexico, and Sri Lanka (4th or 5th depending on the sample). It ranked third in Poland. Meaning and purpose was relatively high ranked across the board, topping the list in one survey (Mexico) and placing second in three additional surveys (Sri Lanka, Poland, and the U.S.). Character and virtue was the top-ranked domain for employees in Sri Lanka, Poland, and the U.S.; it was ranked 2nd in Cambodia, and 3rd in Mexico and China. The domain of financial and material stability always ranked last, even in the U.S., while happiness and life satisfaction ranked 5th in Sri Lanka, Mexico, China, and Poland (4th in Cambodia and the U.S.). The most heterogeneous ranks were observed for the mental and physical health domain, which was ranked second best in Mexico and China, and second to last in Cambodia (3rd in the U.S. and Sri Lanka, and 4th in Poland).

## Discussion

Our findings on six domains of well-being measured with two aggregated indexes (SFI and FI; VanderWeele 2017) were drawn from surveys of factory workers in six countries including Mexico, Sri Lanka, Cambodia, China, Poland, and the U.S. We suggested that a focus on the well-being of factory workers is especially important because this group generates the economic productivity that supports other groups, particularly in less developed countries where presence of this group of workers usually exemplifies a shift from more traditional agricultural jobs to more modern and higher income positions.

We found that financial and material stability ranked last in all examined samples with Mexico scoring the lowest. Mexican respondents ranked the meaning and purpose domain highest of all the domains of well-being and their mean score was higher than in other countries. Relative to the other nations in our study, Mexican respondents in the Gallup World Poll (2017) also reported the highest ranking for purpose, although that survey used a rather different measure framed in terms of motivation to accomplish personal goals and enjoyment of daily activities. Yet, the contrast between our Mexican sample—with its low level of financial security but high levels of meaning and purpose—and our U.S. results— with higher financial security, but lower meaning and purpose— is quite striking.

Moving on to China, concerns have been expressed about the impact of rapid economic development and drastic cultural shifts on well-being in this part of the world (Shek, 2010). For instance, the traditional Chinese culture praises collectivism and interdependence, and the most commonly identified determinants of well-being have been social relationships and health (Rudy et al., 2007). The infusion of the individualistic and egalitarian values over the past few decades, however, has led to certain ideological shifts, and individualistic factors have increasingly shaped the assessment of subjective well-being in the Chinese population (Steele & Lynch, 2013). Nevertheless, our findings add to the evidence that despite the cultural shifts, the traditional collectivist orientation within Chinese culture remains dominant, and the social relationship component still appears to be a highly valued aspect of well-being among the Chinese. Likewise, traditional Chinese culture promotes a peaceful mindset and expressions of intense hedonic emotions are often discouraged (Lu, 2001), which may help with understanding why happiness ranked relatively low (5th ) among the Chinese participants. Furthermore, rapid economic development in modern China has been accompanied by a massive urbanization and migration process, which led to a drastic increase in housing and education prices as well as widening income inequality (Chen et al., 2011; Wang et al., 2017). Thus, it is perhaps not surprising that financial security had the lowest ranking among all well-being domains in the Chinese sample. It is, however, quite interesting that the levels of overall well-being were higher among the Chinese participants as compared to the U.S. and Polish samples. This is somewhat contrary to prior work in which individuals from East Asian countries often report lower levels of subjective well-being compared to their counterparts from Western nations, perhaps partly due to the influences of their contrasting cultural values on self-assessment and reporting (i.e., cultural response bias, see Krys et al., 2023; Lai et al., 2013). However, many prior surveys have not considered the full range of well-being domains assessed in this study.

According to the World Happiness Report (WHR; Helliwell et al., 2019), happiness in Sri Lanka is quite low relative to other countries around the world (130th out of 156 nations), and below each of the countries included in this study. It was confirmed by our results. Our sample of Sri Lankan participants had the lowest mean score for happiness relative to the other five samples and they ranked happiness in fifth place out of the six domains of well-being. Consistent with low happiness, the suicide rate in Sri Lanka is double that of the U.S., three times higher than in China, and over six times that of Mexico (WHO 2018). Although Sri Lanka is often excluded from international studies of well-being, analysis by the UK-based think tank New Economics Foundation (http://happyplanetindex.org/countries/sri-lanka) suggests that despite low well-being (118th place, out of 140 countries), Sri Lanka does exhibit relatively good life expectancy, a small ecological footprint, and only a moderate level of inequality. We also note that the standard deviations reported in Table 2 were highest in Sri Lanka, which indicates that Sri Lankan respondents were highly divergent in their well-being. More research is needed to build on the findings of this study and develop a better understanding of well-being of production workers in Sri Lanka. It is important to note that our survey data were collected prior to the onset of the current economic crisis in this country and that findings are likely to be different with more recent data (Marris, 2022).

Financial security in Poland not only ranked lowest among the six well-being domains but also the mean score was much lower than in the U.S., China, Sri Lanka, and even Cambodia. This was an unexpected outcome, especially for a developed economy and an OECD country. There is, however, some evidence that financial aspirations of Polish population have been increasing recently and outpacing the growth of incomes (Panek et al., 2015). The growing income aspirations may translate into dissatisfaction with current level of incomes, increased financial insecurity, and lead to expectations of even faster improvement in financial position in the future. Poles are generally very pessimistic about their financial situation (in 2015 only 33% of Poles declared being content or very content with their financial situation; Czapiński & Panek 2015, p. 206). Polish factory workers also provided a low assessment of their health relative to the other domains of well-being (ranked 5th ) and the lowest compared to other examined groups of workers. Low assessment in this domain is partially substantiated by low objective measures of health (Weziak-Bialowolska, 2014). But it is probably even more affected by the so-called Polish ‘culture of complaining’ (Dolinski, 1996), which translates into a subjective sense of underperforming in some domains but not others. Indeed, one of the domains for which complaining is socially acceptable in Poland is health (Wojciszke, 2014). The opposite is true for the social relations, which may partially explain the comparatively higher position—it was ranked 3rd —of this domain in the Polish well-being assessment.

Cambodians ranked close social relationships highest and their mean scores across all domains were higher than those in other samples. In fact, also their FI mean as well as SFI mean were the highest across all samples. Cambodia is not frequently included in international studies of well-being, so we do not have a strong basis for explaining these patterns. However, estimates from the World Health Organization (2018) suggest that life expectancy is not especially high in Cambodia, which might shed some light on why mental and physical health was ranked fifth out of the six domains by our Cambodian respondents. The Gallup World Poll (2017) also reveals that respondents from Cambodia were the least likely to respond favorably to an assessment of physical health, compared with respondents from other countries in our study. The low score for financial and material stability (ranked last among the six domains; only Poland and Mexico had lower means) is consistent with our expectations in light of both low gross domestic product and indicators originating from other survey data (Weziak-Bialowolska et al., 2019b).

In the recent World Happiness Report with data like our samples, collected prior to the COVID-19 pandemic, Twenge (2019) suggested that “the years since 2010 have not been good ones for happiness and well-being among Americans” (p. 88). The lead author of the 2019 WHR report identified a central finding: “What stands out about the happiest and most well-connected societies is their resilience and ability to deal with bad things… [their] high social capital, where people are connected…” (Helliwell, quoted in Hetter 2019). Social support—measured with the binary item, “If you were in trouble, do you have relatives or friends you can count on to help you whenever you need them, or not?”—was the only highly significant predictor of life satisfaction, positive affect, and negative affect worldwide (Helliwell et al., 2019, p. 20). Continuing a long-term decline, the U.S. dropped to 19th place in overall happiness in the survey, partly because of addictions to substances and technology and because “social connections are weakening” (Hetter, 2019). In fact, one of the authors of the report explained that the downward trajectory in well-being is a function of the U.S. being “a mass-addiction society” (Sachs, 2019, p. 124). Other research estimates that roughly half of “the U.S. adult population suffers from maladaptive signs of an addictive disorder” (Sussman et al., 2011, p. 3) to substances or behaviors, which has drawn increased attention to the relationship between declining social connections as a cause of addiction, and thus a root cause of declining well-being (Lee et al., 2019). The U.S. does rank highly in one category (income, in 10th place globally), but not on any other measure of well-being; it is only in 37th place for social support, the WHR’s measure of social connectedness.

In our surveys, factory workers from the U.S. sample scored higher than workers in all other countries on financial and material stability, but lower on close social relationships (the lowest score compared to workers in Mexico, China, Cambodia, Poland, and Sri Lanka). The financial component of well-being was, however, still ranked last. As abundant social science research concluded, comparatively high financial scores are not necessarily associated with high scores on other domains of well-being. Declining well-being and rising addiction, along with co-occurring mental disorders such as depression and anxiety, are related to the withering of community (Hari, 2018; Lee et al., 2019; Putnam, 2000). Lack of strong social connections decreases well-being and increases “social pathology,” particularly in individualistic nations like the U.S. (Diener & Suh, 1999, p. 443). Since U.S. respondents rate social connectedness as the least important well-being domain (Lee et al., 2021), and they also experience low levels of connectedness compared to workers abroad, this may suggest that they may have adapted their personal preferences to an unhealthy, materialistic, addiction-prone social environment. The consequences of such “adaptive preferences” are becoming apparent. Technology addicted U.S. youths now spend an average of six hours of their leisure time each day on digital media, with nearly half being online “almost constantly,” despite the inverse relationship between many such activities and happiness (Shakya & Christakis, 2017; Twenge, 2019, p. 89).

Our study is not without limitations. First, we did not have a nationally representative sample from each country. Our samples of workers were diverse. Participants in some of them were much younger and more likely to be female than the general population. However, to increase the comparability of results, we applied post-stratification weights. Second, the results of this study should be interpreted in light of known challenges with regard to comparing survey responses across cultures. Future research might compare how variations in mean well-being scores could be attributable to cultural differences in response patterns to one or more items on the SFI or FI. It would also be helpful to compare our subjective findings with objective measures of well-being and quality of life, such as political freedom, economic opportunity, and participation in regenerative environmental practices. Nevertheless, our results provide more detailed benchmarking on six domains of well-being and two composite measures of well-being among production workers than studies that have used more generalizable samples but assessed fewer domains of well-being.

## Conclusion

Being in line with a number of calls for valuing, examining and improving worker well-being (Schill, 2017; Tamers et al., 2019), this study shows that the level of various domains of well-being of production workers varies across organizations (and possibly countries), even if these organizations are engaged in improving worker’s well-being and have implemented a worker well-being program. Therefore, our study indicates that promotion of general worker well-being initiatives might be ineffective. Instead, tailored programs taking into account specificity of targeted groups of employees might emerge as a valuable business resource helping organizations to retain workforce and increase productivity.

## Data Availability

The dataset is not publicly available.

## Footnotes

1 https://betterwork.org.

2 Domain 1: Happiness and Life Satisfaction: (1) Overall, how satisfied are you with life as a whole these days? (2) In general, how happy or unhappy do you usually feel? Domain 2: Mental and Physical Health: (3) In general, how would you rate your physical health? (4) How would you rate your overall mental health? Domain 3: Meaning and Purpose: (5) Overall, to what extent do you feel the things you do in your life are worthwhile? (6) I understand my purpose in life; Domain 4: Character and Virtue: (7) I always seek to do what is good and right in all circumstances, even when it is difficult, and I always act accordingly; (8) I am always able to give up some happiness now for greater happiness later; Domain 5: Close Social Relationships: (9) I am content with my friendships and relationships; (10) My relationships are as satisfying as I would want them to be; Domain 6: Financial and material stability: (11) How much do you worry about being able to meet your normal monthly expenses? (12) How often do you worry about safety, food, or housing?
